# Editorial: Cross-Talk Mechanisms of Wnt/Beta-Catenin Signaling Components with TLR-Activated Signaling Molecules in the Inflammatory Response

**DOI:** 10.3389/fimmu.2017.01396

**Published:** 2017-10-27

**Authors:** Víctor M. Baizabal-Aguirre

**Affiliations:** ^1^Centro Multidisciplinario de Estudios en Biotecnología, Facultad de Medicina Veterinaria y Zootecnia, Universidad Michoacana de San Nicolás de Hidalgo, Morelia, México

**Keywords:** inflammation, Wnt/beta-catenin, TLR, NF-κB, dendritic cells, *Mycobacterium*, infection

**Editorial on the Research Topic**

**Cross-Talk Mechanisms of Wnt/Beta-Catenin Signaling Components with TLR-Activated Signaling Molecules in the Inflammatory Response**

Molecules and signaling pathways involved in an inflammatory response have been subjects of numerous original and review articles in the last decade. At the beginning and for many years, scientists were mainly interested in deciphering the intricate events involved in triggering and maintaining inflammation. Such studies led to the establishment of NF-κB as one of the main transcription factors that regulates the expression of functionally related genes (i.e., TNFα, IL-1β) involved in controlling inflammation, cell survival, and cancer (1). Efforts to elucidate the molecular events leading to NF-κB activation have identified the widely accepted canonical and non-canonical signaling pathways initiated by similar, but specific ways, depending on the context and type of cell (2).

An uncontrolled cell proliferation and the tumor microenvironment characterized by cells secreting pro-inflammatory cytokines, mostly promoted by NF-κB activity, are undoubtedly the most characteristic hallmarks of all types of cancers (3). During a chronic inflammatory process or during the formation, maintenance, and growth of tumors, NF-κB plays a prominent role. One of the key signaling pathways inducing the expression of genes related to cell division is the Wnt/beta-catenin that is mediated by the Wnt glycoproteins and the transcriptional coactivator beta-catenin. A tight link between the tumor inflammatory microenvironment and cell proliferation, mediated by NF-κB and Wnt/beta-catenin, has been found in different cell types and contexts. Up to date, a cross-talk between these two signaling pathways has been identified in different cell types and physiological settings. The topics of the review articles included in this special issue highlight the current evidence on the interconnection between NF-κB and Wnt/beta-catenin in neuroinflammation [Alzheimer disease (AD)], autoimmunity, cancer, and infection by *Mycobacterium*.

The work by Ma and Hottiger is a very well detailed overview of the different molecules and mechanisms that reciprocally regulate the activities of NF-κB and Wnt/beta-catenin. The authors start with a description of the canonical NF-κB and Wnt/beta-catenin signaling pathway and then concentrate the discussion on the alternative mechanisms used by NF-κB to positively or negatively control the transcriptional activity of beta-catenin and the mechanisms used by beta-catenin to positively or negatively modulate the activity of NF-κB in inflammation, and inflammation-associated diseases such as cancer. Importantly, they conclude that positive or negative control of one signaling pathway over the other is cell and context-dependent and propose that new therapies against cancer should be focused on controlling inflammation and cell proliferation simultaneously.

The AD is a neuroinflammatory disorder of the central nervous system in which the β-amyloid peptide (Aβ) forms aggregates. The article review by Zolezzi and Inestrosa gives a clear account on the possible interrelationship between pro-inflammatory genes expressed by the TLR/NF-κB and Wnt/beta-catenin signaling pathways in the AD. These authors point out that the production of proinflammatory cytokines, like IL-1β, IL-6, IL-12, TNFα, the enzymes cyclooxygenase 2, and the inducible nitric oxide synthase are likely mediated by astrocytes, microglia, neurons, and oligodendrocytes. Interestingly, in a normal brain, the synthesis of these proinflammatory factors is counterbalanced by the expression of anti-inflammatory genes, such as TGFβ and IL-10; however, in AD, proinflammatory gene expression is predominant since Aβ causes a chronic inflammatory condition. This unbalance is perhaps due to the failure of the anti-inflammatory control exerted by the canonical Wnt/beta-catenin components on the TLR-dependent NF-κB activation as it is discussed in the review.

During the last few years, a number of studies have highlighted the role of dendritic cells (DCs) in the regulation of immune responses in various pathological conditions. The review by Suryawanshi et al. discusses the current knowledge on the function of Wnt/beta-catenin signaling in the induction of immune tolerance in homeostatic tissues, autoimmunity, and oncogenesis. An important conclusion drawn by the authors, after discussing many reports on DC roles in different diseases, such as autoimmune encephalomyelitis, rheumatoid arthritis, psoriasis, cancer, and others, is that inhibition of Wnt/beta-catenin activity induces an important reduction of inflammation. In particular, they point out that in the central nervous system a sustained activation of this pathway restrains inflammation, stimulates neuroprotection, and promotes neurogeneration. Finally, but not less important, a clear picture of the tolerogenic role of Wnt/beta-catenin in the gut through a modulatory effects of DCs is also provided.

An increasing number of reports has documented the modulation of Wnt/beta-catenin signaling in different bacterial infections (4). Infection caused by *Mycobacterium tuberculosis*, an intracellular pathogenic Gram-positive bacterium, has been a major burden of humanity with millions of cases per year, being one of the leading cause of death worldwide. The review by Brandenburg and Reiling, puts in perspective the major advances in our understanding of various Wnt (Wnt3a, Wnt5a, and Wnt6) and Frizzled (FZD1) genes function, the upregulation of FZD1 by IFNγ, and the relationship of Wnt/beta-catenin with TLR (TLR2 and TLR4) signaling during *M. tuberculosis* lung infection. Most importantly, they discussed the major contribution of these molecular components inside the granuloma and surrounding tissues.

Another excellent review included in this special issue is the review of Villaseñor et al. on the interconnection between Wnt/beta-catenin and *M. tuberculosis*. These authors emphasize that *M. tuberculosis* induces the secretion of proinflammatory cytokines (i.e., TNFα, IL-6, and IL-1β) from macrophages at early stages of the infection, while at later stages, anti-inflammatory cytokine (i.e., TGFβ and IL-10) expression predominates, which contributes to mycobacterium survival and granuloma formation. Moreover, in addition to a detailed account of Wnt3, Wnt5, and Wnt6 on mycobacterium infection, a noteworthy mention on NOD2 and a variety of miRNAs is included, giving a wider picture of the regulatory mechanisms developed in infections caused by *M. tuberculosis*.

In light of the current experimental evidence on the molecular programs that control an inflammatory response, one would expect major breakthroughs based on the interrelation and integration of signaling pathways such as canonical TLR-IKK-NF-κB and Wnt/beta-catenin. A deep understanding of the regulatory mechanisms among these signaling pathways will be critical to design drugs aimed at effectively controlling inflammation by affecting different protein interactions that are fundamentally altered in chronic and degenerative diseases.

## Author Contributions

VMB-A edited all the article reviews and wrote the editorial of the Research Topic.

## Conflict of Interest Statement

The author declares that the research was conducted in the absence of any commercial or financial relationships that could be construed as a potential conflict of interest.

## References

[1] Ben-Neriah Y Karin M Inflammation meets cancer, with NF-κB as the matchmaker. Nat Immunol (2011) 12:715–23. 10.1038/ni.2060 21772280

[2] Vallabhapurapu S Karin M Regulation and function of NF-κB transcription factors in the immune system. Annu Rev Immunol (2009) 27:693–733. 10.1146/annurev.immunol.021908.132641 19302050

[3] Hanahan D Weinberg RA Hallmarks of cancer: the next generation. Cell (2011) 144:646–74. 10.1016/j.cell.2011.02.013 21376230

[4] Silva-García O Valdez-Alarcón JJ Baizabal-Aguirre VM The Wnt/β-catenin signaling pathway controls the inflammatory response in infections caused by pathogenic bacteria. Mediators Inflamm (2014) 2014:310183 10.1155/2014/310183 25136145 PMC4127235

